# HCMV-IE2 promotes atherosclerosis by inhibiting vascular smooth muscle cells’ pyroptosis

**DOI:** 10.3389/fmicb.2023.1177391

**Published:** 2023-05-10

**Authors:** Guixin Ma, Zhongjie Yu, Fulong Nan, Xianjuan Zhang, Shasha Jiang, Yunyang Wang, Bin Wang

**Affiliations:** ^1^Department of Pathogenic Biology, School of Basic Medicine, Qingdao Medical College, Qingdao University, Qingdao, Shandong, China; ^2^Department of Special Medicine, School of Basic Medicine, Qingdao Medical College, Qingdao University, Qingdao, Shandong, China; ^3^Department of Endocrinology and Metabolism, The Affiliated Hospital of Qingdao University, Qingdao, Shandong, China

**Keywords:** HCMV, IE2, VSMCs, atherosclerosis, pyroptosis

## Abstract

Atherosclerosis is still the main cause of death in developed and developing countries. Vascular smooth muscle cells (VSMCs) death disorder is a key pathogens of atherosclerosis. During the early stage of human cytomegalovirus (HCMV) infection, immediate early protein 2 (IE2) is critical in regulating its host cell death to ensure HCMV replication. Abnormal cell death induced by HCMV infection contributes to the development of numerous diseases, including atherosclerosis. Hitherto, the underlying mechanism of HCMV involved in the progression of atherosclerosis is still unclear. In this study, the infection models *in vitro* and *in vivo* were constructed to explore the pathogenesis of HCMV-related atherosclerosis. Our results indicated that HCMV could contribute to the progression of atherosclerosis by enhancing the proliferation, invasion, and inhibiting the pyroptosis of VSMCs under inflammatory conditions. Meanwhile, IE2 played a key role in these events. Our present research revealed a novel pathogenesis of HCMV-related atherosclerosis, which might help develop new therapeutic strategies.

## 1. Introduction

Atherosclerosis is a chronic progressive inflammatory vascular disease and the leading cause of death worldwide (Kong et al., 2022). Atherosclerosis is the process of plaque formation, including lipids, debris, and various cells in the vascular intima (Basatemur et al., 2019). Among the numerous pathogenic factors, VSMCs are key participants in all processes of atherosclerosis. The abnormal death and proliferation of VSMCs promote the development of atherosclerosis by accelerating plaque formation (Li et al., 2022). Previous studies have found that virus infection contributes to the progression of atherosclerosis by regulating the proliferation of VSMCs (Yonemitsu et al., 1997). However, the underlying mechanisms are yet to be elucidated.

HCMV is a β-herpesvirus, more than 90% of the general population is an HCMV carrier (Clement and Humphreys, 2019). HCMV infection disrupts homeostasis by affecting cell death and proliferation (Babu et al., 2014; Song et al., 2018) and then causes multiple diseases, including atherosclerosis (Liao et al., 2014). More seriously, HCMV can trigger life-threatening diseases in immunosuppressed patients (de Jong et al., 1998). Thus, it is urgent to elucidate the pathogenic mechanism of HCMV and discover novel strategies for anti-HCMV treatment.

Pyroptosis is a newly discovered programmed cell death caused by pro-inflammation and characterized by cell swelling, the protrusion of large bubbles from the plasma membrane, and cell lysis (Xu et al., 2018; Qian et al., 2021). Zeng et al. (2019). report that pyroptosis widely occurs in atherosclerosis initiation, progression, and complications. Therefore, it may be an effective treatment strategy for atherosclerosis by regulating pyroptosis.

This study aimed to investigate the effects of HCMV infection on the development of atherosclerosis. Using *in vitro* and *in vivo* models, we found that HCMV could enhance the proliferation, invasion, and inhibit the pyroptosis of VSMCs, and then promote the development of atherosclerosis. HCMV-derived IE2 played a key role in the pathological process of atherosclerosis. Our study was expected to provide clues for searching for novel therapeutic strategies for atherosclerosis.

## 2. Materials and methods

### 2.1. Cell culture and treatment

VSMCs were kindly presented by Professor Yu (Institute for Translational Medicine, Qingdao University). Cells were cultured in DMEM (Gibco, 11,965,092) with 10% fetal bovine serum (TransGen, FS401-02) and in a humidified 5% CO_2_ incubator at 37°C. HCMV laboratory strain AD169 was kindly presented by Professor Wang (Department of Pathogenic Biology, Qingdao University) and VSMCs were infected by HCMV at MOI = 20. Manidipine dihydrochloride (MND, Selleck, S2482) was used to repress IE2 expression at 80 μM. When the cultured cells reached approximately 80% confluency, they were treated with lipopolysaccharide (LPS, Solarbio, L8880).

### 2.2. CCK-8 assay

Cell activity and cytotoxicity were detected using a cell counting kit-8 (CCK-8) (Abclonal, RM02823). The experiments were performed according to the instructions of the manufacturer.

### 2.3. Immunoblotting

Immunoblot was carried out as previously described (Li et al., 2002). Briefly, the cells were lysed for 30 min on ice in RIPA lysis buffer containing a protease inhibitor cocktail. The samples were subjected to 12% SDS-PAGE and transferred to PVDF membranes. Blots were probed with primary antibodies anti-IE2 (Abcam, ab53495, 1:1,000), anti-NLRP3 (Affinity, BF8029, 1:1,000), anti-GSDMD (Affinity, AF4012, 1:1,000), anti-IL1β (Affinity, BF8021, 1:1,000), anti-Caspase1 (Affinity, AF5418, 1:1,000) and anti-β-actin (TransGen, HC201-01, 1:2,000) at 4°C overnight. After being washed with TBST, the goat anti-mouse IgG (H + L), HRP conjugate (TransGen, HS201-01, 1:2,000) or goat anti-rabbit IgG (H + L), HRP Conjugate (TransGen, HS101-01, 1:2,000) were added. Omni-ECL™ Femto Light Chemiluminescence Kit (Epizyme, SQ201) was used to visualize antigen–antibody complexes, and the density of blots was quantified by Image J.

### 2.4. The IE2 plasmid construction

The expression plasmid for IE2 was generated by amplifying the corresponding cDNA from HCMV-infected VSMCs by PCR with Phanta Max Super-Fidelity DNA Polymerase (Vazyme, P505-d1). Next, we cloned it into the pcDNA3.1 expression vector using ClonExpress Ultra One Step Cloning Kit (Vazyme, C115-01). Lipofectamine 3000 (Invitrogen^™^, L3000015) was used for plasmid transfection. The procedures followed the kit instructions.

### 2.5. Wound healing

Wound healing was carried out as others previously described with slightly modified (Tan et al., 2021). A sterile 200 μL pipette tip was used to scratch a constant width area, and cells were stimulated with or without LPS in a 6-well plate for 24 h. Wound closures were monitored and photographed by using a microscope (Olympus, BX50). The width of wound healing at 0 and 24 h was measured by ImageJ.

### 2.6. Cell invasion assays

Matrigel (BD Biosciences, 356,234) was added to the upper chamber. Cells cultured with or without LPS were resuspended in 200 μL serum-free medium and then loaded into the upper chamber. A complete medium (600 μL) was added to the lower chamber. After 24 h, the invasive cells onto the underside were fixed with 4% paraformaldehyde, stained with 0.1% crystal violet (Solarbio, G1063), and counted under microscopy. Invasion ability was analyzed through the number of crystal violet-positive cells.

### 2.7. Mouse model

Ethical Statement: All animal experiments were performed according to the guidelines of the Animal Welfare and Research Ethics Committee of Qingdao University.

*IE2* transgenic mice were constructed by Shanghai Model Organisms Center, Inc. Mouse was randomly divided into four groups: WT-control, *n* = 5; IE2-control, *n* = 5; WT-model, *n* = 5; IE2-model, *n* = 5. The control group was given a normal diet without LPS stimulation. The model groups were given a 1/2 high-fat (Medicine, MD12033) diet and 0.5 μg/g LPS by weekly intraperitoneal injection. After 12 weeks, samples were collected and analyzed. The transgenic mice construction, identification, and experimental workflow are shown in Supplementary Figure 1.

### 2.8. Serum lipids tests

Blood samples were collected for serum lipids tests. Samples were placed at room temperature for 30 min and then centrifuged at 4°C, 3,000 g for 30 min. The supernatants were transferred into new tubes for examination. The tests were performed in Servicebio Co., Ltd. (Wuhan, China).

### 2.9. Oil red O staining

Lipid accumulation of the thoracoabdominal aorta was determined by oil red O staining (Servicebio, G1015). Aorta was fixed with 4% paraformaldehyde for at least 24 h, then washed with PBS. After soaking 60% isopropyl alcohol for 3 s, the aorta was stained by oil red O at 37°C for 30 min. The excess oil red O was removed by using 60% isopropyl alcohol. Lastly, the samples with a scale were photographed and quantified.

### 2.10. H&E staining

Xylene and decreasing concentrations of ethanol were used for slice dewaxing. Then, the slices were stained with a hematoxylin and eosin staining kit (Servicebio, G1005). After staining, slices were dehydrated through increasing concentrations of ethanol and xylene. Slices were sealed by glycerin jelly and then imaged using a microscope (Olympus, BX50).

### 2.11. Hoechst 33342/PI staining

The slices of the aorta were double stained with a Hoechst 33342/PI kit (Solarbio, CA1120) according to the manufacturer’s instructions. Briefly, dewaxed slices were double-stained with Hoechst 33342 and PI at 4°C for 40 min. Subsequently, the stained slices were observed with a fluorescence microscope (Olympus, BX50).

### 2.12. Statistical analysis

The results are expressed as mean ± SEM of at least three independent experiments. The statistical comparison among different groups was performed by one-way analysis of variance (ANOVA) for multiple comparisons. Statistical analyses were performed with GraphPad Prism 8.0 (GraphPad Software, Inc., San Diego, CA). *p* < 0.05 was considered statistically significant.

## 3. Results

### 3.1. LPS leads to VSMCs pyroptosis

**Figure 1:**
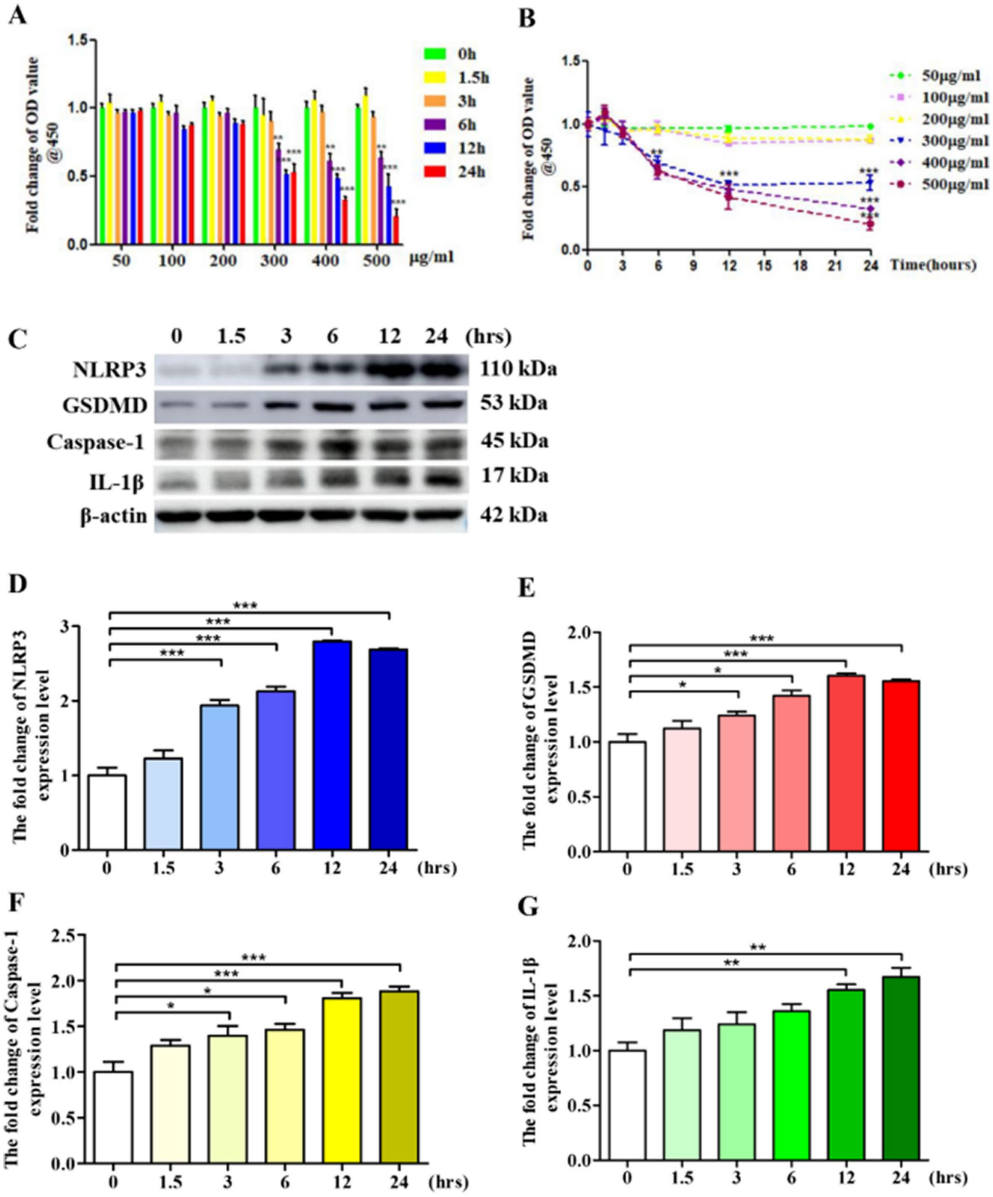
LPS leads to VSMCs pyroptosis. **(A,B)** 1 × 10^5^ VSMCs were treated with different concentration LPS for different time. Cell activity was detected by using a CCK-8 kit. **(C)** 1 × 10^6^ VSMCs were treated with 300 μg/mL LPS for 0, 1.5, 3, 6, 12, and 24 h, respectively. Western blotting detected the expression level of pyroptosis markers. **(D–G)** Statistical analysis of NLRP3, GSDMD, Caspase-1, and IL-1β, respectively. The data was expressed as the mean ± SEM of three independent experiments. The statistical comparison among different groups was performed by ANOVA for multiple comparisons. Statistical analyses were performed with GraphPad Prism 8.0. *p < 0.05, **p < 0.01, ***p < 0.001.

Pyroptosis is a programmed cell death triggered by inflammation. Pyroptosis is involved in the occurrence and development of various diseases (Yu et al., 2021). LPS is a bacterial-derived inflammatory inducer, often used to establish various inflammation-related disease models (Chen et al., 2021). In this study, we used LPS to construct a VSMCs pyroptosis model. Firstly, we observed the effect of different concentrations of LPS on VSMCs cell activity at different time points and determined the optimum concentration and time point of LPS. The results showed that 300 μg/mL, 12 h and 24 h could signifivantly inhibit the activity of VSMCs (Figures 1A,B). Next, we examined the expression levels of pyroptosis markers, key proteins in the pyroptosis signaling pathway (Hou et al., 2021). As shown in Figures 1C–G, after stimulation with LPS, the expression levels of pyroptosis markers, including NLRP3, GSDMD, Caspase-1, and IL-1β, were significantly elevated time-dependently, and in 24 h group the expression quantity of pyroptosis markers was highest. Because of its significantly inhibit the activity of VSMCs and relatively high inductivity of pyroptosis, 300 μg/mL, 24 h was selected for further study.

### 3.2. HCMV blocks VSMCs pyroptosis

**Figure 2:**
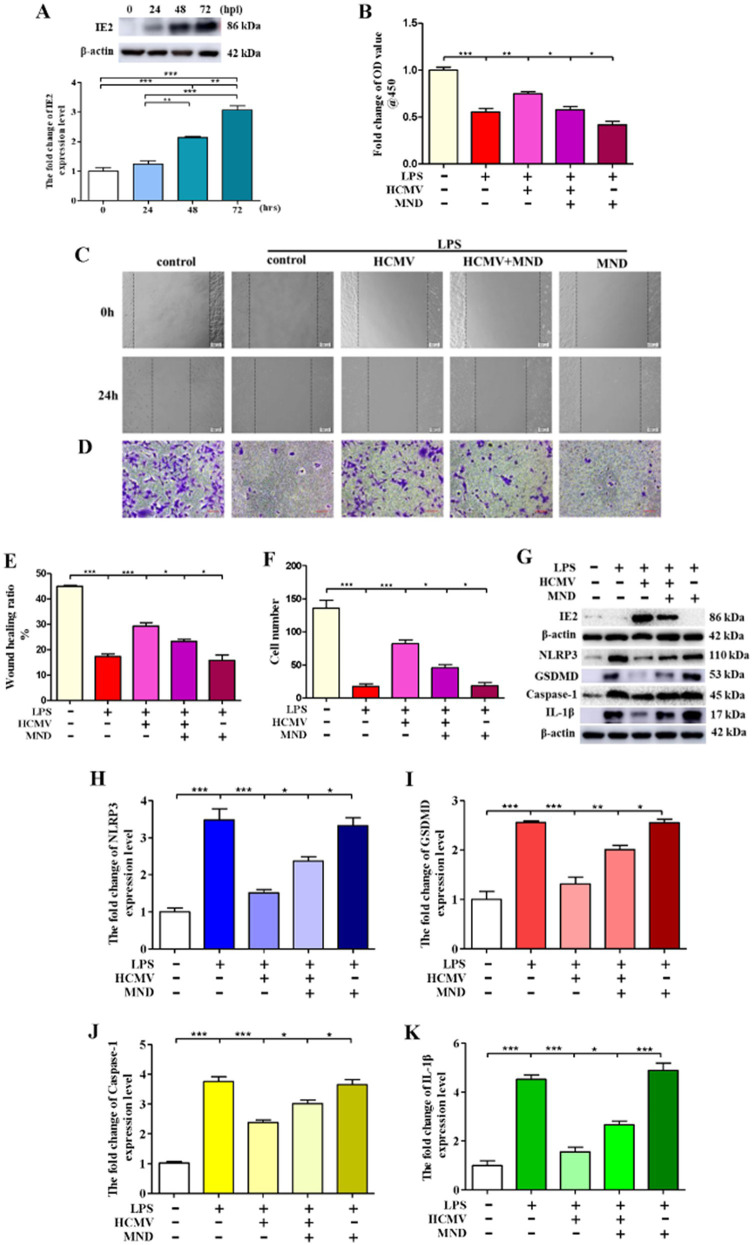
HCMV blocks VSMCs pyroptosis. 1 × 10^6^ VSMCs were infected by HCMV at MOI = 20 for 24, 48, and 72 h. The infected VSMCs was treated with 80 μM MND for 24 h to inhibit the expression of IE2. **(A)** The expression of IE2 was detected by western blotting. **(B)** 1 × 10^5^ VSMCs were treated with 300 μg/mL LPS for 24 h. Cell activity was detected by using a CCK-8 kit. **(C,D)** Cell invasion ability was measured by wound healing and cell invasion assays, bar = 100 μm. **(E,F)** Statistical analysis of wound healing and cell invasion assays. **(G)** 1 × 10^6^ VSMCs were treated with 300 μg/mL LPS for 24 h. Western blotting detected the expression level of IE2 protein and pyroptosis markers. **(H–K)** Statistical analysis of NLRP3, GSDMD, Caspase-1, and IL-1β, respectively. The statistical comparison among different groups was performed by ANOVA for multiple comparisons. Statistical analyses were performed with GraphPad Prism 8.0. The data were expressed as the mean ± SEM of three independent experiments. *p < 0.05, **p < 0.01, ***p < 0.001.

HCMV is widely prevalent worldwide and regulates the occurrence and development of various diseases, including atherosclerosis (Yu et al., 2021). Atherosclerosis is a major risk factor for cardiovascular and cerebrovascular diseases, the leading cause of death worldwide (Lusis, 2000). Previous studies have reported that HCMV infection involves atherosclerotic plaque rupture and myocardial infarction (Prochnau et al., 2011). However, the exact mechanisms of atherosclerosis induced by HCMV infection remain unclear. To investigate the mechanisms, we proved that HCMV could infect VSMCs (Figure 2A), and HCMV infection significantly blocked VSMCs’ death induced by LPS (Figure 2B). Moreover, HCMV promoted VSMCs invasion (Figures 2C–F), the main inducement of atherosclerosis (Huang et al., 2020). Mechanistic research found that HCMV infection could inhibit VSMCs’ pyroptosis (Figures 2G–K). These data indicated that HCMV infection inhibited VSMCs death and promoted VSMCs invasion by blocking VSMCs pyroptosis.

### 3.3. HCMV-derived IE2 plays a key role in the regulation of VSMCs pyroptosis

Our previous studies demonstrated that IE2 is the main pathogenic factor of HCMV (Mu et al., 2019). In this research, to explore the role of IE2 in HCMV-regulated VSMCs pyroptosis, we used MND to repress the expression of IE2 (Mercorelli et al., 2018). As shown in Figure 2B, inhibition of the expression of IE2 significantly reduced cell viability and remarkedly blocked VSMCs’ invasion (Figures 2C–F). Moreover, after IE2 deprivation, VSMCs pyroptosis induced by LPS significantly increased (Figures 2G–K).

**Figure 3:**
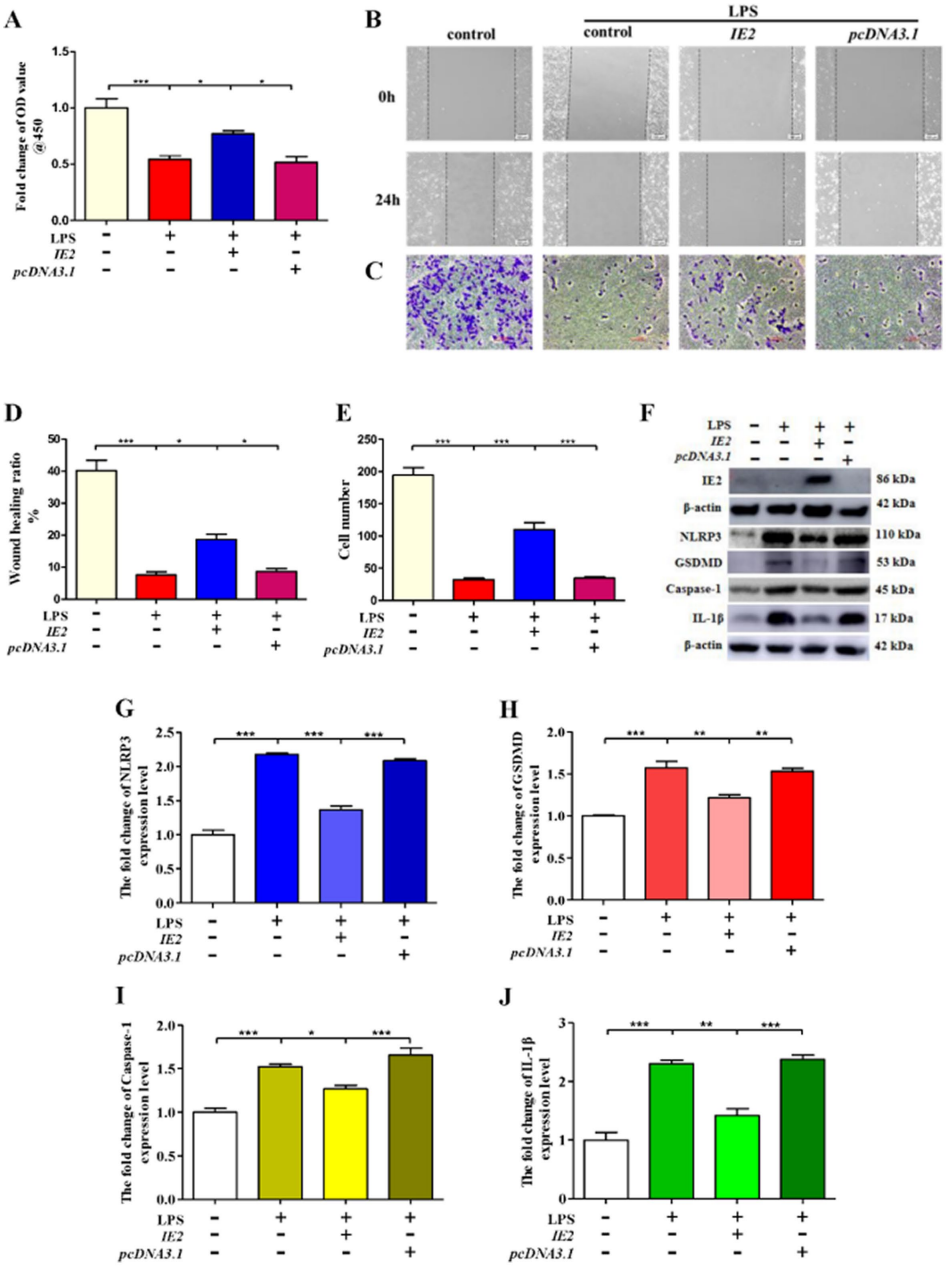
IE2 plays a key role in the regulation of VSMCs pyroptosis. 1 × 10^6^ VSMCs were transfected with 2 μg *IE2* plasmid by using Lipofectamine 3,000. VSMCs were treated with LPS on the second day after transfection *IE2* plasmid. **(A)** 1 × 10^5^ VSMCs were treated with 300 μg/mL LPS for 24 h. Cell activity was detected by using a CCK-8 kit. **(B,C)** Cell invasion ability was measured by wound healing and cell invasion assays, bar = 100 μm. **(D,E)** Statistical analysis of wound healing and cell invasion assays. **(F)** 1 × 10^6^ VSMCs were treated with 300 μg/mL LPS for 24 h. Western blotting detected the expression level of IE2 protein and pyroptosis markers. **(G–J)** Statistical analysis of NLRP3, GSDMD, Caspase-1, and IL-1β, respectively. The statistical comparison among different groups was performed by ANOVA for multiple comparisons. Statistical analyses were performed with GraphPad Prism 8.0. The data were expressed as the mean ± SEM of three independent experiments. *p < 0.05, **p < 0.01, ***p < 0.001.

To further confirm the role of IE2 in regulating VSMCs pyroptosis, we constructed an *IE2* overexpression plasmid. In contrast to IE2 deprivation, overexpression of IE2 in VSMCs could reverse the reduction of cell viability caused by LPS (Figure 3A). Under inflammatory conditions created by LPS, IE2 significantly promoted VSMCs’ invasion (Figures 3B–E). Besides, overexpression IE2 observably reduced VSMCs pyroptosis caused by LPS (Figures 3F–J). Our data demonstrated that IE2 played a key inhibitory effect on VSMCs pyroptosis induced by LPS and further facilitated VSMCs invasion.

### 3.4. IE2 promotes atherosclerosis by inhibiting the aortic cells’ pyroptosis in mice

**Figure 4:**
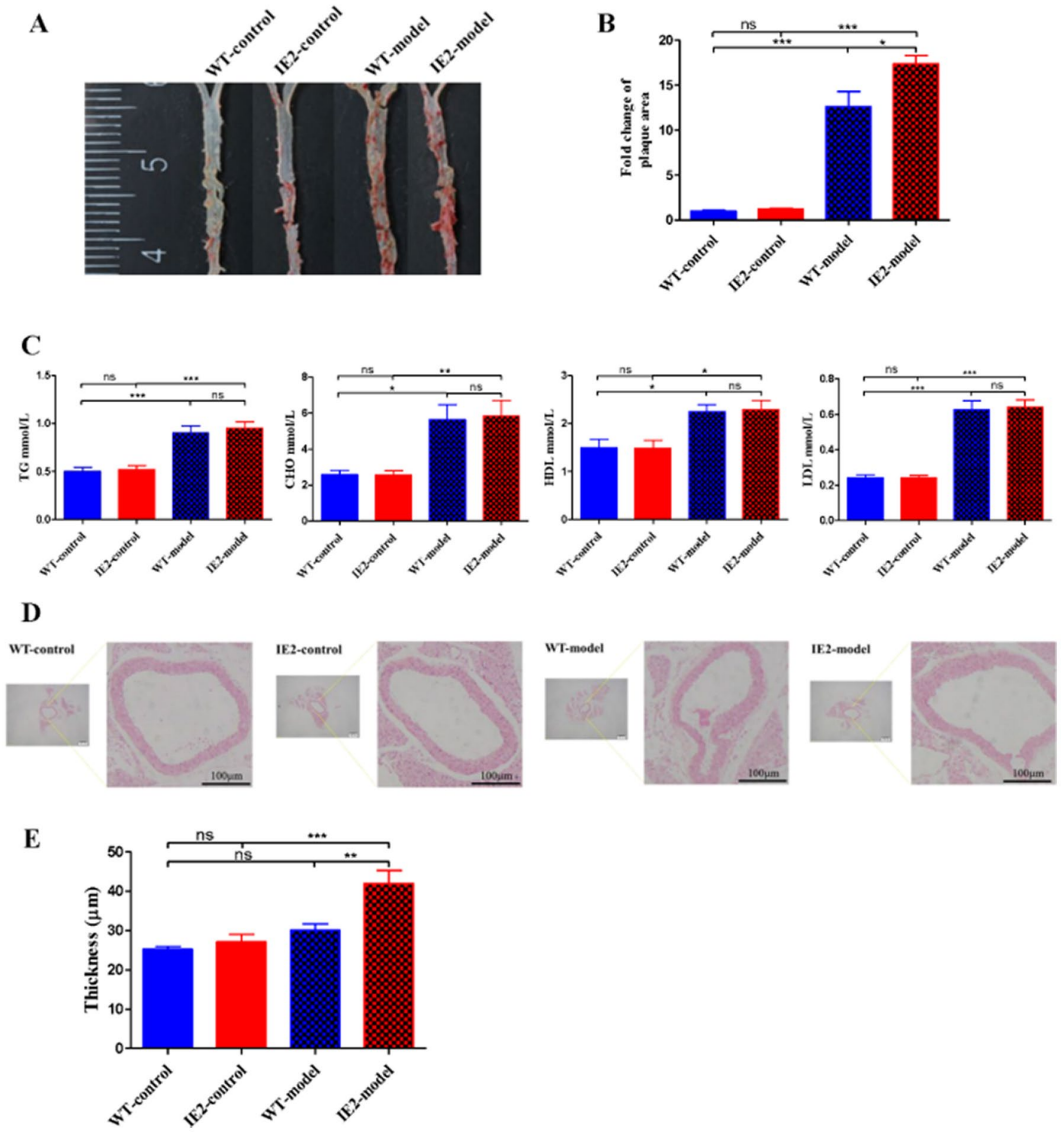
IE2 promotes the development of atherosclerosis. Mouse was randomly divided into four groups: WT-control, *n* = 5; IE2-control, *n* = 5; WT-model, *n* = 5; IE2-model, *n* = 5. The control group was given a normal diet without LPS stimulation. The model group was given a 1/2 high-fat diet and 0.5 μg/g LPS by weekly intraperitoneal injection. **(A)** Atherosclerotic plaques were detected by using oil-red O staining. **(B)** Statistical analysis of plaque area. **(C)** Statistical analysis of serum lipids tests. **(D)** HE staining of the aorta, bar = 100 μm. **(E)** Statistical analysis of aorta thickness. The statistical comparison among different groups was performed by ANOVA for multiple comparisons. Statistical analyses were performed with GraphPad Prism 8.0. The data were expressed as the mean ± SEM, *n* = 5. *p < 0.05, **p < 0.01, ***p < 0.001. ns = no statistical significance.

**Figure 5:**
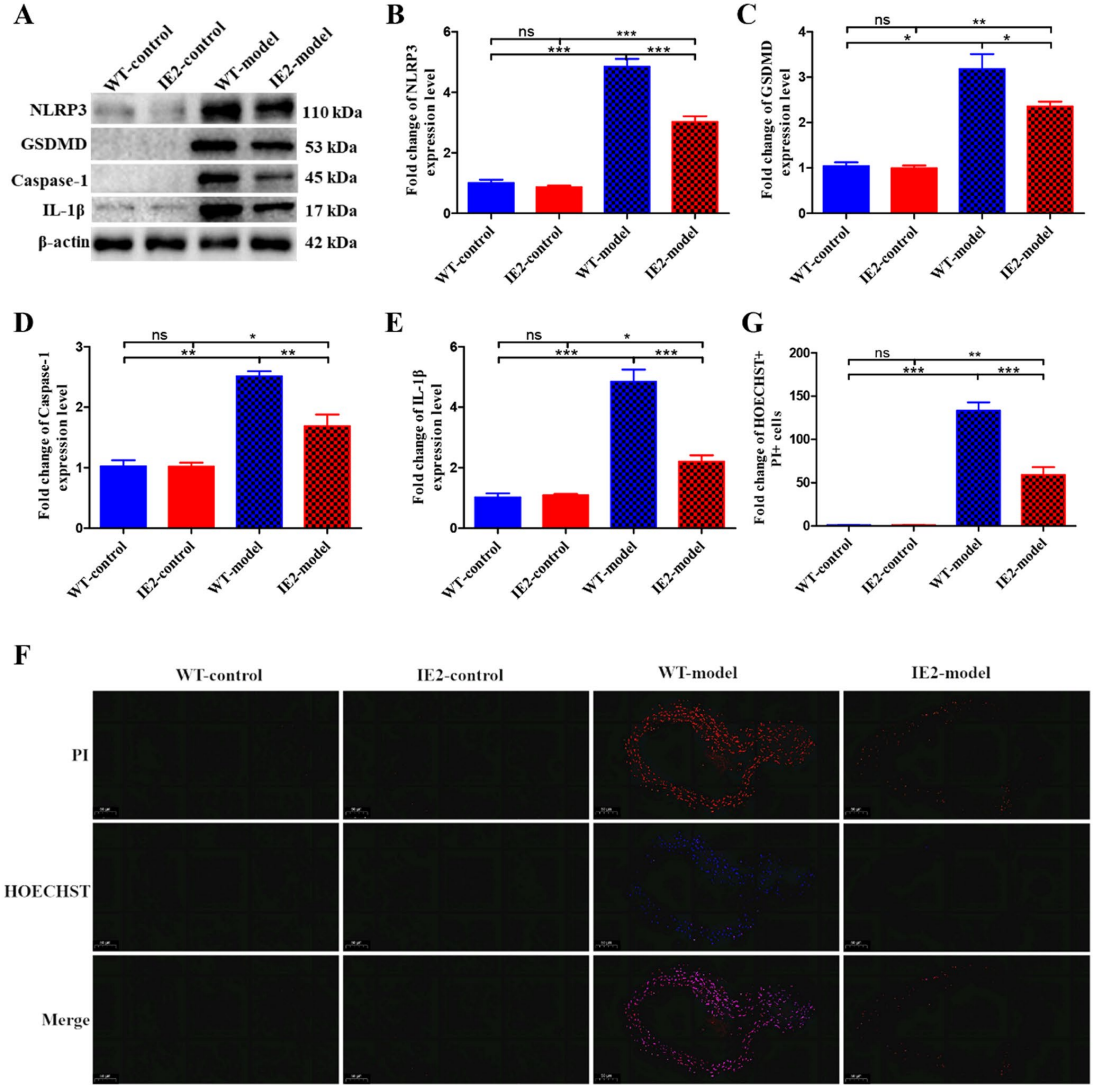
IE2 blocks VSMCs pyroptosis *in vivo*. **(A)** Western blotting detected the expression level of pyroptosis markers. **(B–E)** Statistical analysis of NLRP3, GSDMD, Caspase-1 and IL-1β, respectively. **(F)** Pyroptosis of vascular cells was observed by using Hoechst 33342/PI staining, bar = 50 μm. **(G)** Statistical analysis of Hoechst 33342 and PI double positive cell population. The statistical comparison among different groups was performed by ANOVA for multiple comparisons. Statistical analyses were performed with GraphPad Prism 8.0. The data were expressed as the mean ± SEM, *n* = 5. *p < 0.05, **p < 0.01, ***p < 0.001. ns = no statistical significance.

*IE2* transgenic mice were constructed to test the role of IE2 in the development of atherosclerosis *in vivo*. IE2 significantly increased lipid accumulation and atherosclerotic plaque formation in the thoracoabdominal aorta (Figures 4A,B). Serum lipids tests indicated that compared with the IE2-control group, the increased lipid accumulation and atherosclerotic plaque formation was not caused by increased dyslipidemia (Figure 4C) but maybe induced by the unusual accumulation of vascular cell and the thickened artery wall (Figures 4D,E). Mechanistic studies revealed that IE2 could reduce pyroptosis of aortic cells (Figures 5A–E). Moreover, using Hoechst/PI double staining to detect pyroptosis (Hu et al., 2021) also confirmed that IE2 blocked the pyroptosis of aortic cells (Figures 5F,G). These data indicated that IE2 promoted atherosclerosis development *in vivo* by enhancing vascular cell accumulation and artery wall thickening.

## 4. Discussion

Atherosclerosis is a multifactorial disease that affects the normal lives of hundreds of millions of people and results in heavy social and economic burdens. However, the pathogenesis is still not fully understood, especially in viral-associated atherosclerosis. In this study, we revealed that HCMV infection could increase cellular activity, inhibit pyroptosis and promote invasion of VSMCs under inflammatory conditions.

*IE2* is a key gene involved in the occurrence and development of numerous HCMV-related diseases (Yu et al., 2021). Yonemitsu et al. reported that *in vivo IE* gene transfer led to neointimal thickening, which may play a role in the progression of atherosclerosis (Yonemitsu et al., 1997). Nevertheless, the detailed molecular mechanism has yet to be reported. In the present study, our data indicated that *IE2* could inhibit pyroptosis and contribute to the accumulation of vascular cells in the abdominal aorta, accelerating the development of atherosclerosis. In addition, Tanaka et al. revealed that IE2 protein blocked p53-mediated apoptosis and induced smooth muscle cell accumulation, thereby contributing to restenosis and atherosclerosis (Tanaka et al., 1999). However, IE2 protein contributes to the development of atherosclerosis by regulating pyroptosis of vascular cells has yet to be reported previously. In this study, our results revealed that IE2 protein could inhibit the pyroptosis of vascular cells by decreasing the expression of NLRP3, GSDMD, Caspase-1, and IL-1β. Moreover, IE2 protein also promoted VSMCs’ invasion and accumulation in the abdominal aorta, ultimately resulting in atherosclerosis.

Certain aspects of our current study should be improved. Samples from patients with atherosclerosis should be tested in the next research to determine whether most of them are HCMV positive. Furthermore, VSMCs markers should be used in animal samples to provide more relevant data. Despite its limitations, our present study reveals a novel mechanism of viral-associated atherosclerosis, and these data may provide a potential therapeutic strategy for atherosclerosis.

## Data availability statement

The original contributions presented in the study are included in the article/Supplementary material, further inquiries can be directed to the corresponding authors.

## Ethics statement

The animal study was reviewed and approved by the Animal Welfare and Research Ethics Committee of Qingdao University.

## Author contributions

GM, ZY, and BW: project administration and writing and editing. ZY, GM, YW, FN, XZ, and SJ: experiment. GM, YW, FN, and ZY: analyzing data. ZY and GM: original draft preparation. ZY and BW: funding acquisition. All authors have read and agreed to the published version of the manuscript.

## Funding

This research was supported by the Shandong Provincial Science and Technology Foundation (grant no. 2019JZZY011009), Qingdao Municipal Science and Technology Foundation (grant no. 20-2-3-4-nsh), National Key Research and Development Program of China (grant no. 2018YFA0900802), Qingdao Postdoctoral Application Research Project (grant no. RZ2100001326), and Shandong Provincial Natural Science Foundation (grant no. ZR2021QH254).

## Conflict of interest

The authors declare that the research was conducted in the absence of any commercial or financial relationships that could be construed as a potential conflict of interest.

## Publisher’s note

All claims expressed in this article are solely those of the authors and do not necessarily represent those of their affiliated organizations, or those of the publisher, the editors and the reviewers. Any product that may be evaluated in this article, or claim that may be made by its manufacturer, is not guaranteed or endorsed by the publisher.

## Abbreviations

VSMCs: Vascular smooth muscle cells
HCMV: Human cytomegalovirus
IE2: Immediate early protein 2
hpi: Hours of post-infection
MND: Manidipine dihydrochloride
