# Supplementary material for: HCMV-IE2 promotes atherosclerosis by inhibiting vascular smooth muscle cells’ pyroptosis
Source: Front Microbiol. 2023 May 10;14:1177391. doi: 10.3389/fmicb.2023.1177391 (PMC10206012; doi:10.3389/fmicb.2023.1177391)
Supplement: Supplementary file 1 [file Image_1.pdf]

Supplementary figure.

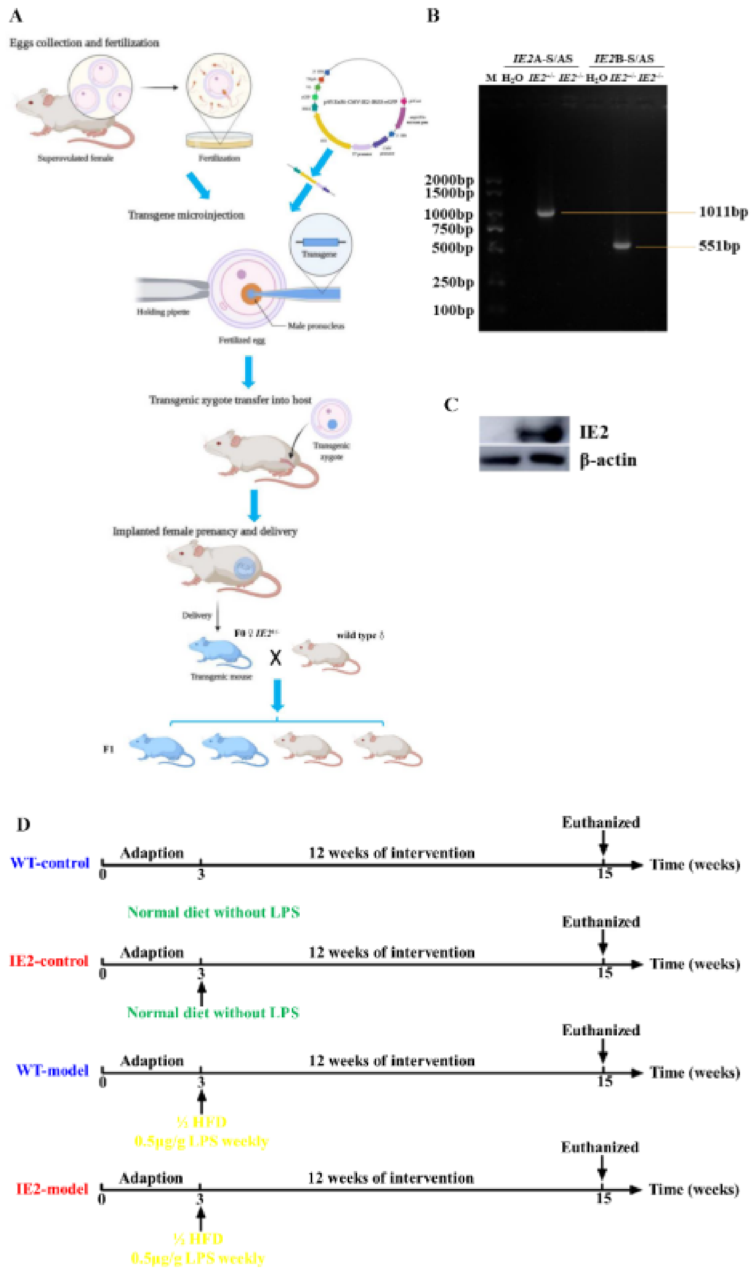

Supplementary figure 1.

A. The workflow of *IE2* transgenic mice construction.

B, C. The identification of *IE2* transgenic mice by PCR and western blotting.

D. The timeline of experiments in vivo.
